# Role of Pentoxifylline and Sparfloxacin in Prophylaxis of Spontaneous Bacterial Peritonitis in Cirrhotic Patients

**DOI:** 10.1155/2014/595213

**Published:** 2014-03-06

**Authors:** Tarek Mohammed Mostafa, Osama Mohamed Ibrahim, Gamal Abd El-Khalek Badra, Mahmoud Samy Abdallah

**Affiliations:** ^1^Department of Clinical Pharmacy, Faculty of Pharmacy, Tanta University, Tanta 31527, Egypt; ^2^National Liver Institute, Menoufiya University, Shebeen El Kom 32111, Egypt

## Abstract

This study was directed to evaluate the role of sparfloxacin and pentoxifylline in the prophylaxis of spontaneous bacterial peritonitis in cirrhotic patients. Forty cirrhotic patients with ascites were included in the study. Patients were randomized into four groups in a blind fashion; each group consists of ten patients. Group one received ciprofloxacin (control group), group two received sparfloxacin, group three received pentoxifylline, and group four received a combination of sparfloxacin and pentoxifylline. Treatment duration was six months. Serum TNF-**α** level was the primary inflammatory marker of the study to evaluate the effect of the used medications. In group two, TNF-**α** level showed a statistically significant decrease in comparison with group one (*P* = 0.001), while in group three, TNF-**α** level showed nonsignificant difference in comparison with the control group (*P* > 0.05). In addition, group four showed a statistically significant decrease in TNF-**α** level compared to the other three groups (*P* < 0.05). The finding from our study indicates that sparfloxacin as well as pentoxifylline could be used in prophylaxis of spontaneous bacterial peritonitis. Combination of sparfloxacin and pentoxifylline showed some of synergism which may be useful in decreasing emergence of resistant strains.

## 1. Introduction

Spontaneous bacterial peritonitis (SBP) is a common and severe complication of cirrhotic patients having ascites with a prevalence rate between 10 and 30% characterized by spontaneous infection of ascitic fluid which occurs in the absence of any infection or perforation of intra-abdominal organs [1]. Approximately 20% of patients are already infected at the time of admission and nearly 50% develop an infection during hospitalization [2]. Patients with the greatest risk for the development of SBP are those who have recovered from the first episode. In these patients, the recurrence rate is very high; the probability of developing a new episode of SBP ranges from 40% to 70% within the first-year followup [3, 4]. SBP is now associated with in-hospital mortality rates ranging from 20% to 40% [5]. Furthermore, mortality rates one and two years after an episode of SBP are reported to be 50–70% and 70–75%, respectively [6]. However, mortality after SBP is improved owing to early diagnosis and prompt treatment with empiric antibiotics. Bacterial translocation (BT) and migration of viable microorganisms from the intestinal lumen to the mesenteric lymph nodes and other extraintestinal sites have been postulated as the main mechanism in the pathogenesis of SBP [7–9]. Translocation of the enteric organisms to mesenteric lymph nodes is increased in patients with advanced cirrhosis and is reduced by selective intestinal decontamination [10]. According to European Association for the Study of the Liver (EASL) guidelines [11], the administration of prophylactic antibiotics reduces the risk of recurrent SBP. Norfloxacin (400 mg/day, orally) is the treatment of choice. Alternative antibiotics include ciprofloxacin (750 mg once weekly, orally) or cotrimoxazole (800 mg sulfamethoxazole and 160 mg trimethoprim daily, orally).

Sparfloxacin is a broad spectrum antibiotic, active against varieties of bacteria that are considered predisposing agents for SBP such as E. coli, Klebsiellae,* Enterobacter aerogenes*,* Shigella*,* Yersinia* pestis, and other Gram-negative microorganisms. In addition, it belongs to third generation fluoroquinolones (FQs) which have better activity against Gram-positive* Cocci* and anaerobes in comparison with ciprofloxacin. The difference in spectrum of activity is largely caused by increased activity against the DNA-gyrase of Gram-positive bacteria, rather than activity against Topoisomerase IV, which is the target in Gram-positive bacteria for the older quinolones [12, 13].

Pentoxifylline, [3,7-dimethyl-1-(5-oxohexyl)xanthine], is a methyl xanthine derivative with a significant protective effect in infection of Gram-negative sepsis and peritonitis in animal models [14]. It was found to have the property to block the inflammatory action of interleukin-1 (IL-1) and TNF-*α* on neutrophils and thus was able to diminish the tissue damage caused by neutrophils in morbid conditions like septic shock [15]. Pentoxifylline prevents bacterial translocation after intestinal obstruction in an experimental model as ischemic injury of intestinal mucosa plays a role in pathogenesis of bacterial translocation [16, 17]. In cirrhotic rats with ascites, pentoxifylline as well as norfloxacin reduces intestinal bacterial overgrowth, bacterial translocation, and spontaneous bacterial peritonitis [18]. In addition, it was presented that pentoxifylline, but not norfloxacin, reduces oxidative stress in cecal mucosal [18]. This may explain the expected beneficial role of pentoxifylline in the prevention of bacterial infection in patients with advanced cirrhosis.

Therefore, this research aimed to test new prophylactic therapies against microbes causing SBP. In this context, the role of both sparfloxacin and pentoxifylline as prophylactic therapy for SBP in patients with cirrhosis was investigated.

## 2. Materials and Methods

Forty patients with cirrhosis and ascites who had at least one previous episode of SBP were recruited from National liver Institute, Menoufiya University, Shebin El kom, Egypt. Patients were included in a randomized, blind, and controlled study. Diagnosis of cirrhosis was based on clinical, biochemical, and/or histological criteria. Inclusion criteria were age >18 and <80 years and participants gave their written informed consent. The protocol was approved by the ethics committee of National liver Institute, Menoufiya University, Shebin El kom, Egypt, with Institutional Review Board (IRB) protocol number 0063/2012. The diagnosis of SBP was confirmed if the ascitic fluid polymorphonuclear cell (PMN) count was greater than 250 mm^3^ with or without positive culture and by absence of an intra-abdominal source of infection. Ascitic fluid cultures were performed using the conventional culture method and via inoculating 10 mL of fluid in aerobic and anaerobic blood culture bottles at the bedside.

Exclusion criteria included active gastrointestinal bleeding, encephalopathy (>grade 2), hepatocarcinoma or other malignancies, and allergy to quinolones.

At admission, patients were divided into four groups. Group one received ciprofloxacin 750 mg/week orally as prophylactic therapy (*n* = 10) (Ciprobay 750 mg tablet, Hikma pharma S.A.E under license of Bayer-Schering pharma, Germany) according to European Association for the Study of the Liver guidelines, group two received sparfloxacin 200 mg tablet (Spara 200 mg tablet, Global Nabi pharmaceuticals, Egypt) every other day for 10 days and then twice/week (*n* = 10), group three received pentoxifylline 400 mg tablet (Trental 400 mg SR tablet, Sanofi Aventis Egypt under license of Sanofi Aventis, Germany) once daily for 10 days and then twice/week, and group four received combination of sparfloxacin and pentoxifylline as scheduled above in groups two and three. The treatment was continued for six months. The etiology of cirrhosis for all patients encountered in this study was viral infection. Liver function was evaluated using Child-Pugh Classification [19]; all patients were classified as Child C.

At enrollment, physical examination, liver and renal function tests, sodium level, red and white blood cells count, platelets count, hemoglobin level, prothrombin time, and serum TNF-*α* concentrations were measured at baseline, three and six months after treatment.

Patients were followed up closely every month with careful assessment to rule out any complications such as fever, abdominal pain, or other symptoms or signs of infection. Study medication was discontinued in the case of recurrent SBP that represents end point of the trial. The drugs used in the study were withdrawn in patients suffering from other complications such as gastrointestinal bleeding or encephalopathy and receiving the standard treatment in each case.

About 10 mL of blood was taken from each patient by sterile venipuncture, without frothing and after minimal venous stasis using disposable syringes. About 3 mL of venous blood was delivered in a vacutainer serum separator tube. Immediate centrifugation at 3000 rpm to avoid contamination of the sample with erythrocyte arginase was done, and then serum samples were used for testing liver and renal function tests. (All kits used for biochemical analysis were supplied from Siemens Healthcare Diagnostics Products Gmbh, Germany, Cat. No. OUHP 29). The optical density for all these parameters was measured using Shimadzu UV-PC 1601, spectrophotometer, Japan.

### 2.1. Measurement of Liver Function Parameters

Serum alanine aminotransferase (ALT) and serum aspartate aminotransferase (AST) were measured spectrophotometrically using kinetic method [20, 21], serum bilirubin level (total and direct) was measured spectrophotometrically using colorimetric (Diazo) method [22], measurement of serum albumin concentration was determined spectrophotometrically using modified bromocresol green colorimetric method [23], and prothrombin time was determined by coagulation method [24].

### 2.2. Measurement of Renal Function Parameters

Blood urea nitrogen was determined spectrophotometrically using enzymatic (fixed rate) UV method with urease and glutamate dehydrogenase [25], serum creatinine concentration was determined spectrophotometrically using buffered kinetic Jaffé reaction without deproteinization method [26], and sodium level was determined colorimetrically [27].

### 2.3. Measurement of Hematological Parameters

About 2 mL of venous blood was delivered in a graduated vacutainer plastic tube containing 3.6 mg of potassium-ethylenediaminetetraacetic acid (K-EDTA) for complete blood count (CBC), haemoglobin (Hb) (Sysmex Automated Hematology Analyzer KX-21N,, Japan), white blood cells (WBCs), red blood cells (RBCs), and platelets (PLTs) (Sysmex Corporation, Kobe 651-0073, Japan).

### 2.4. Measurement of TNF-*α*

About 3 mL of venous blood was drawn in EDTA tubes containing the protease inhibitor aprotinin for measurement of TNF-*α*. These tubes were kept refrigerated before blood sample collection. Serum was separated within 30 minutes after blood drawing and kept frozen at −70°C for measurement of TNF-*α* levels using Boster's Human TNF-alpha Elisa kit immunoassay (Boster Biological Technology, LTD, USA) using Biotek Elx 800-UV microtiter plate reader, USA.

### 2.5. Statistical Analysis

Statistical analysis of the data was done considering an alpha error of 0.05 with a 95% confidence interval. Data are presented by mean ± SD. Continuous data were tested using either paired *t*-test or one-way analysis of variance (ANOVA) as required for quantitative variables. Multiple comparisons were done using Tukey's method for all pairwise comparisons (Tukey's HSD). The statistical analysis was performed with IBM^©^SPSS Statistics V20 (SPSS Inc., USA).

## 3. Results

The period of recruitment was from December 2012 to November 2013. At National liver Institute, Menoufiya University, Shebin El kom, Egypt, 65 cirrhotic patients were enrolled in this study. There were 25 patients excluded from this study. (13 patients had hepatocellular carcinoma and 12 patients had severe gastrointestinal bleeding and subsequently died). Only 40 patients were randomized to this study (10 patients in each group). Demographic data of the participants defined as age, sex, weight, smoking, and other systemic disorders such as diabetes and hypertension was demonstrated in Table 1.

Liver and renal function tests, sodium level, complete blood picture, and TNF-*α* at the baseline for the four groups presented by mean ± SD showed nonsignificant difference between groups (ANOVA, *P* > 0.05), therefore, any changes happened after treatment were due to the used medication not due to the individual variations as shown in Table 2. Clinical and laboratory characteristics of patients in the four groups three and six months after treatment presented by mean ± SD were demonstrated in Tables 3 and 4, respectively.

After six months of treatment, group two showed a statistically significant decrease in TNF-*α* level in comparison with the control group (*P* = 0.001) with nonsignificant differences in other laboratory data between it and the control group. They also showed a statistically significant decrease in TNF-*α* level in comparison with group three (*P* = 0.002), while group three showed nonsignificant change in TNF-*α* level compared to the control group (*P* > 0.05). In addition, group four showed a statistically significant decrease in TNF-*α* level in comparison with control group, group two, and group three (*P* = 0.000, *P* = 0.006, and *P* = 0.000), respectively.

For control group, TNF-*α* level three and six months after treatment showed a statistically significant decrease in comparison with its baseline data (paired *t*-test, *P* = 0.01 and *P* = 0.000), respectively. In addition, there was a statistically significant change six months after treatment compared to its level after three months of treatment (paired *t*-test, *P* = 0.001) with decrease of about 14.9%.

For group 2, TNF-*α* level three and six months after treatment showed a statistically significant decrease in TNF-*α* level in comparison with its baseline data (paired *t*-test, *P* = 0.000 and 0.000), respectively, but there was no significant change six months after treatment in comparison with its level after three months of treatment (paired *t*-test, *P* > 0.05).

For group three, TNF-*α* level, three and six months after treatment, showed a statistically significant decrease in TNF-*α* level in relation with its baseline data (paired *t*-test, *P* = 0.000 and 0.005), respectively, but there was no significant change six months after treatment in comparison with three months results (paired *t*-test, *P* > 0.05).

**Figure 1:**
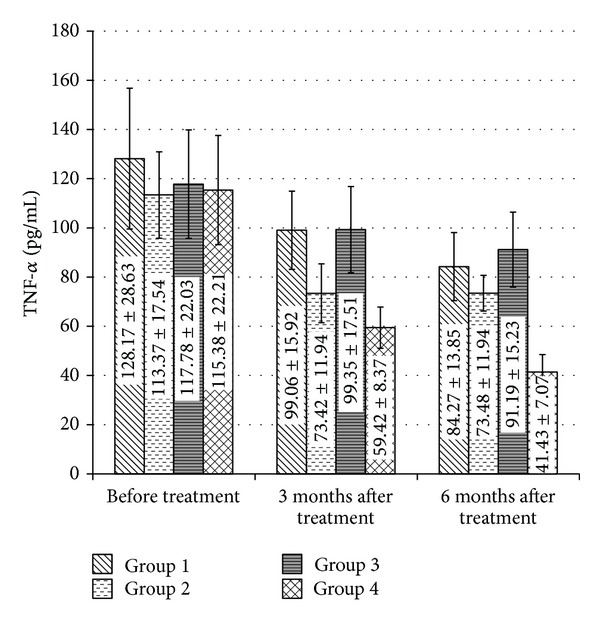
Changes in TNF-*α* level by treatment groups before treatment, three and six months after treatment. Data presented by mean ± SD. TNF-*α* level in the four groups decreases significantly (*P* < 0.5) three and six months after treatment in comparison with its level before treatment.

For group four, TNF-*α* level three and six months after treatment showed a statistically significant decrease in TNF-*α* level in comparison with its baseline data (paired *t*-test, *P* = 0.000 and *P* = 0.000), respectively, with decrease of about 48.4% and 64%, respectively. In addition, there was a statistically significant decrease in TNF-*α* level six months after treatment compared to three months results (paired *t*-test, *P* = 0.024) with decrease of about 30%. The change in TNF-*α* level within the four treatment groups by time is demonstrated in Figure 1.

Serum creatinine level in group three after six months of treatment showed a statistically significant decrease in comparison with the control group (*P* = 0.000). This decrease in serum creatinine level in group three after six months of treatment was also statistically significant compared to group two and group four (*P* = 0.000 and *P* = 0.000), respectively.

**Figure 2:**
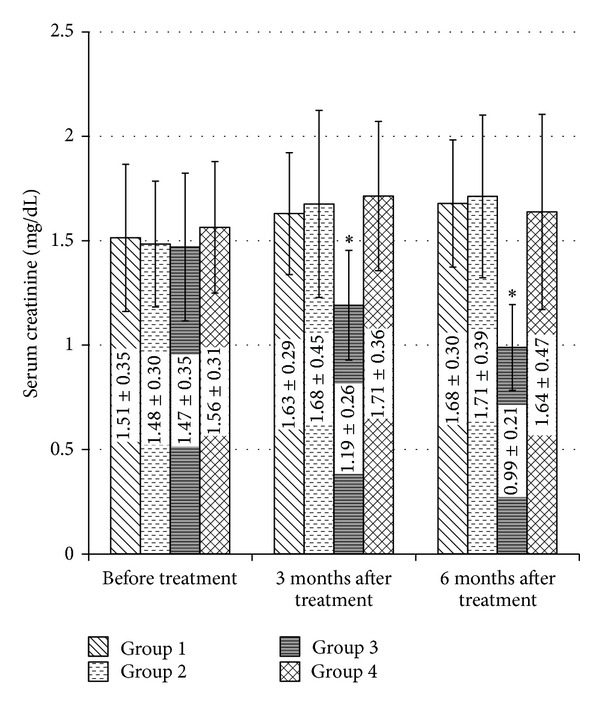
Changes in serum creatinine by treatment groups before treatment, three and six months after treatment. Data presented by mean ± SD. *P < 0.05 in comparison with serum creatinine before treatment.

For group 3, serum creatinine showed a statistically significant decrease three and six months after treatment in comparison with its baseline data (paired *t*-test, *P* = 0.005 and *P* = 0.001), respectively. There was also a statistically significant decrease in serum creatinine six months after treatment in comparison with three months results (paired *t*-test, *P* = 0.036). The change in serum creatinine level within the four treatment groups by time is demonstrated in Figure 2.

Hemoglobin level in group three showed nonsignificant increase in comparison with the control group six months after treatment (*P* > 0.05) with mean 10.28 ± 0.58 g/dL versus 8.80 ± 0.62 g/dL, respectively. This increase in hemoglobin level was statistically significant in relation with group two and group four (*P* = 0.018 and *P* = 0.001), respectively, after six months of treatment.

**Figure 3:**
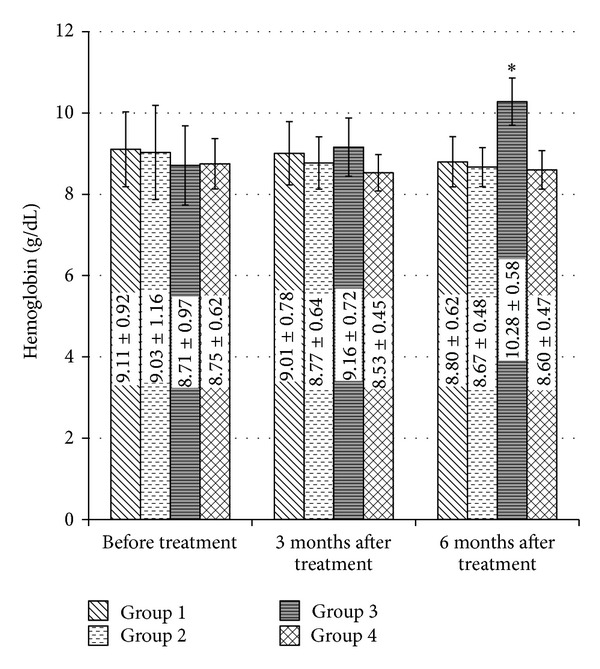
Changes in hemoglobin concentration by treatment groups before treatment, three and six months after treatment. Data presented by mean ± SD. *P < 0.05 in comparison with hemoglobin concentration before treatment.

For group three, hemoglobin level showed nonsignificant increase three months after treatment in comparison with its baseline data (paired *t*-test, *P* > 0.05) with increase of about 5%, but there was a statistically significant increase six months after treatment compared to its baseline data and three months after treatment (paired *t*-test, *P* = 0.000 and *P* = 0.000), respectively, with increase of about 18% and 12.85%, respectively. The change in hemoglobin level within the four treatment groups by time is demonstrated in Figure 3.

## 4. Discussion

This study was the first one that investigates the effect of pentoxifylline and sparfloxacin as a third generation fluoroquinolones antibiotic and a combination of pentoxifylline and sparfloxacin in prophylaxis of SBP. The results of the current study strongly support the efficacy of primary prophylactic therapy in patients with SBP. Sparfloxacin and pentoxifylline significantly reduce the probability of SBP, a common complication in patients with cirrhosis that carry a high mortality rate.

Serum TNF-*α* level was the primary inflammatory marker of the study to evaluate the effect of the used medications. Selection of serum TNF-*α* level based on Goldman and coworkers [28] data shows that bacterial translocation was associated with increased serum TNF-*α*. Bacterial translocation is one of the main events in the pathogenesis of spontaneous bacterial peritonitis [29]. Some of the factors involved in BT are bacterial-dependent (virulence and overgrowth), while others are related to intestinal hypomotility, permeability, mucosal oedema, structural changes in the intestinal wall, and mucosal peroxidation [29]. Selective intestinal decontamination with poorly absorbable antibiotics decreases intestinal bacterial overgrowth (IBO) and BT in experimental and human cirrhosis, with subsequent prevention of SBP [18].

Sparfloxacin showed a statistically significant decrease in TNF-*α* level in comparison with ciprofloxacin. This may be due to broad spectrum activity of sparfloxacin against varieties of bacteria including Gram-negative and Gram-positive bacteria and some anaerobes [12, 13] in comparison with ciprofloxacin especially with increasing frequency of Gram-positive bacteria in spontaneous bacterial peritonitis [30]. The basic mechanisms underlying FQs immunomodulatory activity have not been elucidated in a comprehensive and satisfying manner. Sparfloxacin exerts its immunomodulatory activities by inhibition of dipeptidyl peptidase IV enzyme (DPP IV) in a dose-dependent manner [31]. Sparfloxacin was given as 200 mg every other day for 10 days as an initial dose then twice/week based on prolonged elimination half-life in cases of renal insufficiency after a single oral dose [32]. Also endotoxin, an active component in the outer membrane of the gram negative bacteria, decreases the biliary excretion of sparfloxacin and its glucuronide probably due to impairment of their hepatobiliary transport systems and renal handling [33]. The long elimination half-life could be an advantage, resulting in bactericidal concentrations for prolonged periods, which would make twice/week treatment possible.

Pentoxifylline showed no difference in TNF-*α* level in comparison with ciprofloxacin. This difference in TNF-*α* level may be statistically nonsignificant, but it may be clinically important to keep patients away from antibiotic resistance and adverse effects. The beneficial effect of pentoxifylline on decreasing bacterial translocation is its ability to enhance leukocyte functions. It is well known that translocated bacteria can be cleared by intestinal and mesenteric macrophages [34–36]. Then, translocated bacteria can be cleared by increased mesenteric leukocyte functions mediated by pentoxifylline [37–39]. Pentoxifylline also inhibits the production of TNF-*α* by endotoxin-stimulated monocytes/macrophages at the transcriptional level and is effective in reducing serum TNF-*α* level in mice with endotoxic shock [40], so pentoxifylline as anti-TNF-*α* agent could decrease bacterial translocation as previously mentioned by Goldman and coworkers [28]. Heller and coworkers [41] have shown that pentoxifylline improves bacterial clearance during hemorrhage and endotoxemia and these authors suggested that pentoxifylline could reduce the risk of bacterial infections by attenuating bacterial colonization of organs. Further investigations showed that pentoxifylline potentially affects endotoxin-induced release of TNF-*α* which plays an important role in superantigen-mediated shock [18]. Other beneficial effects of pentoxifylline include improvement in microcirculation that leads to increased bactericidal effect of chemotherapeutic agents [42]. In addition, pentoxifylline promotes physiological changes in fibroblasts resulting in better wound healing [42]. This apparently surprising finding in inhibition of TNF-*α* by pentoxifylline is in disagreement with the previous reported by Lebrec et al., (2010) showing the failure of pentoxifylline to decrease serum TNF-*α* levels in patients with advanced cirrhosis [43].

The decrease in serum creatinine level that happened by pentoxifylline in comparison with ciprofloxacin, sparfloxacin, and combination of pentoxifylline and sparfloxacin may be due to improving the renal microcirculation and hemodynamics by pentoxifylline [44], not due to its effect on TNF-*α* synthesis [45] as demonstrated by Akriviadis et al. This explains the improvement in serum creatinine level by pentoxifylline with increasing the duration of therapy as six months results was better than three months results which independent of its effect on TNF-*α*. This potential primary protective effect of pentoxifylline on renal function is confirmed by its efficacy on prevention of hepatorenal syndrome in severe alcoholic hepatitis patients [46] which occurs in the setting of a decrease in effective arterial blood volume, as indicated by a marked activation of vasoconstrictor systems, and increased serum and ascitic fluid cytokines level [47]. On the other hand, there was no improvement in serum creatinine level in the other three groups. Even patients who received combination of both sparfloxacin and pentoxifylline did not show any improvement in serum creatinine level. This may be due to the side effects of sparfloxacin on the renal function. The increase in hemoglobin level shown by pentoxifylline in comparison with other groups may be due to keeping patients away from antibiotics side effects. In addition, pentoxifylline can improve hemoglobin levels in renal failure patients with erythropoietin-resistant anemia [48].

Pentoxifylline was given as 400 mg once daily for 10 days as an initial dose and then twice/week. The choice of this dosing pattern is based on the hypothesis of the decrease in the total plasma clearance and the increase in the absolute bioavailability of pentoxifylline and its active metabolite by six-eight-fold in cirrhotic patient after oral administration of the sustained-release tablet [49]. In addition, inhibition of these cytokines by pentoxifylline evidently occurs at the transcriptional level and can last for up to five days after the final pentoxifylline dose [50]. Since pentoxifylline prevents intestinal bacterial translocation [51], it could be another promising approach in prophylaxis of spontaneous bacterial peritonitis.

Combination of sparfloxacin and pentoxifylline showed a statistically significant decrease in TNF-*α* level in comparison with the other three groups. This may be due to the synergistic effect between pentoxifylline and fluoroquinolones antibiotics resulting in the inhibition of TNF-*α* as demonstrated by Bailly et al. [52]. The synergistic effect between pentoxifylline and sparfloxacin may be due to improvement of microcirculation by pentoxifylline resulting in increasing the bactericidal effect of chemotherapeutic agents [42]. The decrease in TNF-*α* level was better after six months of treatment than after three months in patients who received combination of pentoxifylline and sparfloxacin in comparison with group two and group three. This may be due to the synergistic effect between pentoxifylline and sparfloxacin. It is possible that the shorter followup in our study (six months) may be responsible for the absence of mortality found in this study. Probably the improvement in survival observed in the current study could be related to the reduction of bacterial translocation and the subsequent amelioration of hemodynamic alterations, reducing the risk of bleeding, encephalopathy and infections.

## 5. Conclusion

According to the data obtained by this study, sparfloxacin could be used in prophylaxis of spontaneous bacterial peritonitis due to its broad spectrum of activity against Gram-positive and Gram-negative bacteria and some anaerobes. Pentoxifylline as tumor necrosis factor inhibitor could be another promising approach reported to hinder BT and to be used as prophylactic therapy agent for spontaneous bacterial peritonitis. Sparfloxacin and pentoxifylline show synergistic effect which may be useful in decreasing emergence of resistant strains. The risk to develop bacterial resistance seems to have a low clinical impact compared to the observed benefit. The efficacy of both sparfloxacin and pentoxifylline in the prophylaxis of SBP in cirrhotics needs further prospective studies on large scale.

## Figures and Tables

**Table 1:** Demographic data of the participants.

Parameters	Group 1	Group 2	Group 3	Group 4	*P* value
Age (years)	50.8 ± 4.917	51.5 ± 4.196	50.5 ± 5.212	51.9 ± 5.087	0.50
Sex (male)	7 (70%)	8 (80%)	7 (70%)	7 (70%)	0.250
Weight (kilograms)	80.6 ± 6.26	80.7 ± 7.16	83.4 ± 5.62	80.75 ± 7.35	0.740
Smoking (%)	2 (20%)	2 (20%)	1 (10%)	2 (20%)	0.250
Diabetes (%)	2 (20%)	1 (10%)	2 (20%)	1 (10%)	0.333
Hypertension (%)	1 (10%)	2 (20%)	1 (10%)	1 (10%)	0.250

*n* = 10 for all groups.

**Table 2:** Selected clinical and laboratory features of patients at baseline.

Parameters	Group 1	Group 2	Group 3	Group 4	*P* value
AST (IU/L)	74.1 ± 16.23	78.1 ± 10.65	75.80 ± 11.47	78.10 ± 10.58	0.838
ALT (IU/L)	52.40 ± 14.27	54.40 ± 11.62	50.10 ± 10.68	49.8 ± 8.84	0.664
BIL-T (mg/dL)	2.46 ± 0.90	2.72 ± 0.77	2.57 ± 0.49	2.16 ± 0.71	0.412
BIL-D (mg/dL)	1.11 ± 0.37	1.31 ± 0.44	1.27 ± 0.44	1.21 ± 0.26	0.690
Albumin (g/dL)	2.55 ± 0.37	2.75 ± 0.35	2.60 ± 0.38	2.52 ± 0.34	0.970
PT (Sec.)	28.4 ± 6.70	28.0 ± 5.52	28.1 ± 4.33	27.6 ± 2.07	0.980
BUN (mg/dL)	71.0 ± 20.83	71.6 ± 20.94	71.4 ± 17.21	70.00 ± 11.81	0.992
s.Cr (mg/dL)	1.51 ± 0.35	1.48 ± 0.30	1.47 ± 0.35	1.56 ± 0.31	0.820
Sodium (mEq/L)	124.30 ± 6.85	126.5 ± 3.84	127.60 ± 5.78	128.1 ± 4.58	0.286
Hemoglobin (g/dL)	9.11 ± 0.92	9.03 ± 1.16	8.71 ± 0.97	8.75 ± 0.62	0.589
RBCs (10^6^/uL)	3.22 ± 0.51	3.39 ± 0.43	3.26 ± 0.41	3.31 ± 0.25	0.795
WBCs (10^3^/uL)	8.72 ± 1.70	8.95 ± 1.39	9.12 ± 1.34	9.23 ± 1.35	0.875
Platelets (10^3^/uL)	77.04 ± 17.75	77.76 ± 8.75	77.76 ± 14.27	78.39 ± 11.17	0.795
TNF-*α* (pg/mL)	128.17 ± 28.63	113.37 ± 17.54	117.78 ± 22.03	115.38 ± 22.21	0.49

Data presented by mean ± SD; AST: aspartate transaminase; ALT: alanine aminotransferase; BIL-T: total bilirubin; BIL-D: direct bilirubin; PT: prothrombin time; BUN: blood urea nitrogen; s.Cr: serum creatinine; RBCs: red blood cells; WBCs: white blood cells; TNF-*α*: tumor necrosis factor alpha; pg/mL: picograms per milliliter.

**Table 3:** Selected clinical and laboratory features of patients 3 months after treatment.

Parameters	Group 1	Group 2	Group 3	Group 4	*P* value
AST (IU/L)	80.4 ± 14. 2	83.5 ± 6.74	76.36 ± 11.51	73.50 ± 9.25	0.679
ALT (IU/L)	55.90 ± 10.29	50.40 ± 8.75	51.3 ± 5.79	52.70 ± 12.56	0.380
BIL-T (mg/dL)	2.36 ± 0.63	2.54 ± 0.64	2.57 ± 0.42	2.19 ± 0.69	0.681
BIL-D (mg/dL)	1.10 ± 0.29	1.13 ± 0.28	1.06 ± 0.22	1.11 ± 0.25	0.927
Albumin (g/dL)	2.64 ± 0.31	2.66 ± 0.22	2.65 ± 0.34	2.60 ± 0.28	0.394
PT (sec.)	29.50 ± 6.02	28.7 ± 6.75	29.7 ± 4.85	29.0 ± 2.45	0.158
BUN (mg/dL)	73.1 ± 15.64	72.1 ± 14.7	65.8 ± 12.23	71.5 ± 11.02	0.333
s.Cr (mg/dL)	1.63 ± 0.29	1.68 ± 0.45	1.19 ± 0.26	1.71 ± 0.36	0.508
Sodium (mEq/L)	127.1 ± 4.79	127.4 ± 4.48	127.90 ± 4.53	127.10 ± 4.79	0.973
Hemoglobin (g/dL)	9.01 ± 0.78	8.77 ± 0.64	9.16 ± 0.72	8.53 ± 0.45	0.797
RBCs (10^6^/uL)	3.39 ± 0.25	3.28 ± 0.37	3.37 ± 0.29	3.33 ± 0.30	0.960
WBCs (10^3^/uL)	7.71 ± 1.29	8.01 ± 1.04	7.84 ± 1.33	8.15 ± 1.35	0.984
Platelets (10^3^/uL)	74.43 ± 12.31	75.33 ± 5.86	74.88 ± 8.90	75.04 ± 6.95	0.995
TNF-*α* (pg/mL)	99.09 ± 15.92	73.42 ± 11.49	99.35 ± 17.51	59.42 ± 8.37	0.000

Data presented by mean ± SD; AST: Aspartate transaminase; ALT: Alanine aminotransferase; BIL-T: Total bilirubin; BIL-D: Direct bilirubin; PT: Prothrombin time; BUN: Blood urea nitrogen; s.Cr: Serum creatinine; RBCs: Red blood cells; WBCs: White blood cells; TNF-*α*: Tumor necrosis factor alpha; pg/mL: picograms per milliliter.

**Table 4:** Selected clinical and laboratory features of patients 6 months after treatment.

Parameters	Group 1	Group 2	Group 3	Group 4	*P* value
AST (IU/L)	79.4 ± 10.08	86.0 ± 7.59	74.85 ± 8.92	82.85 ± 8.92	0.110
ALT (IU/L)	51.6 ± 8.06	51.9 ± 7.5	49.80 ± 10.49	56.8 ± 10.49	0.642
BIL-T (mg/dL)	2.40 ± 0.71	2.64 ± 0.64	2.47 ± 0.69	2.65 ± 0.61	0.767
BIL-D (mg/dL)	1.08 ± 0.35	1.16 ± 0.28	1.03 ± 0.25	1.28 ± 0.38	0.391
Albumin (g/dL)	2.71 ± 0.28	2.65 ± 0.25	2.67 ± 0.24	2.65 ± 0.21	0.012
PT (sec.)	30.9 ± 5.09	31.20 ± 5.25	30.7 ± 4.22	30.4 ± 2.07	0.886
BUN (mg/dL)	70.8 ± 13.77	72.9 ± 18.85	61.00 ± 9.03	72.7 ± 9.10	0.206
s.Cr (mg/dL)	1.68 ± 0.30	1.71 ± 0.39	0.99 ± 0.21	1.64 ± 0.47	0.001
Sodium (mEq/L)	128.4 ± 3.98	127.70 ± 4.64	126.60 ± 6.36	127.20 ± 3.79	0.994
Hemoglobin (g/dL)	8.80 ± 0.62	8.67 ± 0.48	10.28 ± 0.58	8.60 ± 0.47	0.001
RBCs (10^6^/uL)	3.35 ± 0.2	3.22 ± 0.36	3.31 ± 0.34	3.26 ± 0.41	0.903
WBCs (10^3^/uL)	7.45 ± 1.30	7.62 ± 0.91	7.26 ± 0.82	7.20 ± 0.88	0.926
Platelets (10^3^/uL)	73.53 ± 9.45	73.261 ± 9.28	73.07 ± 9.70	73.44 ± 8.66	0.424
TNF-*α* (pg/mL)	84.27 ± 13.85	73.48 ± 7.27	91.19 ± 15.23	41.43 ± 7.07	0.000

Data presented by mean ± SD; AST: aspartate transaminase; ALT: alanine aminotransferase; BIL-T: total bilirubin; BIL-D: direct bilirubin; PT: prothrombin time; BUN: blood urea nitrogen; s.Cr: serum creatinine; RBCs: red blood cells; WBCs: white blood cells; TNF-*α*: tumor necrosis factor alpha; pg/mL: picograms per milliliter.
